# First- and second-line treatment strategies for hormone-receptor (HR)-positive HER2-negative metastatic breast cancer: A real-world study

**DOI:** 10.1016/j.breast.2021.02.015

**Published:** 2021-03-12

**Authors:** Debora Basile, Lorenzo Gerratana, Carla Corvaja, Giacomo Pelizzari, Giorgia Franceschin, Elisa Bertoli, Lorenza Palmero, Diego Zara, Martina Alberti, Silvia Buriolla, Lucia Da Ros, Marta Bonotto, Mauro Mansutti, Simon Spazzapan, Marika Cinausero, Alessandro Marco Minisini, Gianpiero Fasola, Fabio Puglisi

**Affiliations:** a Department of Medicine, University of Udine, 33100, Udine, Italy; b Department of Medical Oncology, Unit of Medical Oncology and Cancer Prevention, Centro di Riferimento Oncologico di Aviano (CRO), IRCCS, 33081, Aviano, Italy; c Department of Oncology, ASUFC Santa Maria Della Misericordia, Udine, Italy

**Keywords:** Metastatic luminal breast cancer, Treatment strategies, CDK4/6 inhibitors, MBC

## Abstract

**Background:**

Endocrine therapy (ET) plus cyclin-dependent-kinases 4/6 inhibitors (CDK4/6i) represents the standard treatment for luminal-metastatic breast cancer (MBC). However, prospective head-to-head comparisons are still lacking for 1st line (L) options, and it is still crucial to define the best strategy between 1st and 2nd L.

**Materials and methods:**

717 consecutive luminal-MBC pts treated between 2008 and 2020 were analyzed at the Oncology Department of Aviano and Udine, Italy. Differences about survival outcomes (OS, PFS and PPS) were tested by log-rank test. The attrition rate (AR) between 1st and 2ndL was calculated.

**Results:**

At 1^st^L, pts were treated with ET (49%), chemotherapy (CT) (31%) and ET-CDKi (20%) while, at 2^nd^L, 33% received ET, 33% CT and 8% ET-CDKi. Overall AR was 10%, 7% for CT, 8% for ET and 17% for ET-CDKi. By multivariate analysis, 1^st^L ET-CDK4/6i showed a better mPFS1 and OS. Moreover, 2^nd^L ET-CDK4/6i demonstrated better mPFS2 compared to ET and CT. Notably, 1^st^L ET-CDKi resulted in higher mPFS than 2ndL ET-CDKi. Intriguingly, 1^st^L ET-CDK4/6i was associated with worse mPPS compared to CT and ET. Secondarily, 1^st^L ET-CDK4/6i followed by CT had worse OS compared to 1^st^L ET-CDK4/6i followed by ET. Notably, none of baseline characteristics at 2^nd^L influenced 2^nd^L treatment choice (ET vs. CT) after ET-CDKi.

**Conclusion:**

Our real-world data demonstrated that ET-CDKi represents the best option for 1^st^L luminal-MBC compared to ET and CT. Also, the present study pointed out that 2^nd^L ET, potentially combined with other molecules, could be a feasible option after CDK4/6i failure, postponing CT on later lines.

## Highlights

• To define the best treatment strategy for 1st and 2nd L is crucial for luminal mBC.
• 1st L CDK4/6i-based therapies represent the standard treatment in luminal mBC.
• This study confirmed the key role of 1st L CDK4/6i on a real-world cohort.
• This study suggested a preeminent role for ET after first-line CDK4/6i.
• The 2nd line ET with other molecules could allow to further postpone CT.

## 1 Introduction

Over the last decade, thanks to a combination of new therapeutic agents and diagnostic strategies, the outcome and management of patients with metastatic breast cancer (mBC) gradually improved both on a survival and quality of life stand point. The combination CDK4/6 inhibitors (CDK4/6i) (i.e Palbociclib, Ribociclib and Abemaciclib) with endocrine therapy (ET, i.e. aromatase inhibitors (AI) or Fulvestrant) has significantly increased objective response rate (ORR) and progression-free survival (PFS) of first- and second-line treatments in patients with hormone receptor positive, HER2 negative (luminal) mBC. Although no head-to-head comparison has been tested in clinical trials, recent systematic reviews and meta-analyses have further supported the efficacy of the combinations in terms of PFS and of overall survival (OS) [[1], [2], [3], [4], [5], [6], [7], [8]].

The growing number of potentially active agents in different treatment lines is increasing the complexity of drug development making the choice of study endpoints even more critical [9]. Indeed, overall survival (OS) has been considered the most relevant outcome measure in mBC trials, being an objective measure of the clinical benefit. However, survival may be influenced by post-protocol treatments and some randomised trials might be underpowered to detect OS differences notwithstanding a benefit in PFS [10,11]. To overcome these limits, recent trials have been designed to detect a clinical benefit in terms of PFS and time-to-treatment progression (TTP), as competing causes of death and further-line treatments may have less impact [11].

A critical, still unmet, need is to improve resistance characterization to ET and CDK4/6i, such as ESR1 genetic and epigenetic alterations, FGFR1 amplification RB1 loss or CCNE1 amplification to anticipate clinical progression and guide subsequent treatment lines [[12], [13], [14], [15], [16]]. As a matter of fact, ET and CDK4/6i resistance might confer to MBC aggressive features such as epithelial to mesenchymal transition that eventually are clinically translated in rapid progression and ultimately in an unfavourable prognosis [15,17]. Furthermore, there is no evidence supporting post CDK4/6i progression decision-making, and different strategies such as CDK4/6i beyond progression, CDK4/6i or ET switch and the addition of other targeted agents, including PI3K inhibitors are being studied.

The aim of this study was to provide real-world treatment patterns data for luminal MBC, and to give hypothesis generation insights of their consequent impact on clinical outcome measures.

## 2 Materials and methods

### 2.1 Study design

This was an observational, multicentricer, retrospective study, that evaluated a consecutive series of 717 patients with luminal mBC. The study was conducted in accordance with the ethical principles deriving from the Helsinki Declaration and the current legislation on Observational Studies (Circular Min. Sal. Of September 6, 2002). The study was approved by the Departmental Review Board and by the Ethics Committee (Protocol number CRO-2019-85).

Data concerning clinico-pathological characteristics, treatments for metastatic disease, and blood tests performed within 30 days of the first- and second-line treatment start, were collected retrospectively. Primary objective of this study was to evaluate the impact first- and second-line of treatment strategies in terms of PFS, post-progression survival [PPS] after first-line, and OS) according to treatment type (chemotherapy, CT; ET alone; ET combined with CDK4/6i). Secondary objectives were to evaluate attrition rate according to first-line treatment, the probability of receiving chemotherapy after CDK4/6i, and factors determining the choice of second-line treatment after CDK4/6i.

PFS was defined as time from first-line therapy start until progression disease or death from any cause. According to the treatment line, PFS was defined as PFS1 and PFS2. PPS was defined as the interval between progression and death or last follow-up.

OS was defined as the time between first-line therapy start and death from any cause.

The attrition rate related to first-line treatment was defined as the proportion of patients who started therapy but who, at the time of disease progression were unable to receive further treatment due to disease progression, death, toxicity, or other clinical conditions.

### 2.2 Study population

All patients had histologically or cytologically confirmed luminal (HR positive/HER2 negative) mBC. They provided informed consent for the use of clinical data that were rendered anonymous for purposes of clinical research, epidemiology, training and disease study.

The study population included consecutive patients treated at the Department of Medical Oncology, National Cancer Institute of Aviano and at the Oncology Department of Udine, Italy, from January 2008 to Janury 2020. Data have been obtained from electronic and paper-chart review according to strict privacy standards.

### 2.3 Statistical analysis

Patients’ clinico-pathological characteristics were summarized through descriptive analysis. Categorical variables were reported as frequency distribution whereas continuous variables were reported as median value and range. Differences in terms of survival outcomes were tested by log-rank test and represented by Kaplan-Meier survival curves. Analyses were stratified by line of treatment and type of treatment received.

A Cox proportional-hazards regression model, including also potential confounders (e.g. age, ET naïve, ECOG PS) was used to calculate hazard ratios (HR) of death, with the corresponding 95% confidence intervals (CI), in the different subgroups of patients identified according treatment. To better evaluate if baseline features at second-line could influence the choice of CT or ET after fist-line CDK4/6i, a logistic analysis was performed.

A two-sided P < 0.05 was considered statistically significant. Statistical analyses will be performed with STATA (StataCorp. (2015) Stata Statistical Software: Release 14.2. College Station, TX: StataCorp LP).

### 2.4 Sample size calculation

The sample size was estimated in order to obtain a good performance of the statistical model for the association between patient and tumor characteristics with outcome measures in the multivariate analysis. The aim of the sampling was the achievement of a good “goodness of fit” from the regression model according to Peduzzi and Concato [18,19]. Therefore, considering 20–50 events per variable (EPV) and a final model with approximately 6–7 variables, more than 350 EPV are necessary to obtain an accurate estimation of the statistical model. In the present study, we observed 575 events for PFS1, 466 for PFS2, and 443 for OS. Therefore, we could define an accurate estimation for the multivariate model.

## 3 Results

### 3.1 Descriptive analysis

The study included 717 women with a diagnosis of luminal mBC who underwent first-line treatment (clinical, pathologic and treatment characteristics are displayed in Table 1). Overall, 29% of patients had a *de novo* mBC, 78% were post-menopausal and 15% had ECOG PS > 1. Moreover, 49%, 30% and 20% had respectively visceral, bone-only and non-visceral disease, while, 26% of cases had ≥3 metastatic sites and 66% had ≥5 metastatic lesions. Tumor burden was, moreover, classified in 3 groups by combining metastatic sites and number of lesions. In particular, group 1 (25%) had <3 metastatic sites and ≤5 lesions, group 2 (45%) <3 metastatic sites and >5 lesions or ≥3 metastatic sites and ≤5lesions, group 3 (23%) had ≥3 metastatic sites and >5 lesions.

**Table 1:** Patient’s characteristics

Variables	N (tot 717)	Frequencies
**Age (years)**		
<45	106	14.78%
45-65	314	43.79%
>65	297	41.42%
**Histotype**		
Ductal	513	71.55%
Lobular	127	17.71%
Other	14	1.95%
Missing	63	8.79%
**Ki-67 on primary tumor**		
<14 %	181	25.24%
≥14 %	430	59.97%
Missing	106	14.78%
**ER**		
≤10 %	25	3.49%
>10 %	594	82.85%
Missing	98	13.67%
**BMI (kg/m**^**2**^**)**		
< 25	263	36.68%
≥ 25	293	40.86%
Missing	161	22.45%
***De novo* metastatic disease**		
No	503	70.15%
Yes	208	29.01%
Missing	6	0.84%
**ET naïve**		
No	271	37.80%
Yes	445	62.06%
Missing	1	0.14%
**CT naïve**		
No	311	43.38%
Yes	405	56.49%
Missing	1	0.14%
**Endocrine responsiveness**		
No	301	41.98%
Yes	276	38.49%
Missing	140	19.52%
**Menopausal status**		
Premenopausal	132	18.41%
Postmenopausal	557	77.68%
Missing	28	3.91%
**Sites - first line**		
Bone only	218	30.40%
No visceral	141	19.67%
Visceral	353	49.23%
Missing	5	0.70%
**Number of sites of metastasis**		
<3	532	74.20%
≥3	184	25.66%
Missing	1	0.14%
**Number of metastatic lesions**		
≤5	191	26.64%
>5	474	66.11%
Missing	52	7.25%
**Metastatic sites score**		
<3 sites and ≤5 lesions	177	24.69%
≥3 sites and ≤5 lesions/ < 3 sites and > 5 lesions	321	44.77%
≥ 3 sites and >5 lesions	166	23.15%
Missing	54	7.53%
**ECOG PS – first line**		
≤ 1	494	68.90%
> 1	108	15.06%
Missing	115	16.04%
**First line therapy**		
CT	224	31.24%
ET	352	49.09%
ET plus CDK 4/6	141	19.67%
**Second line therapy**		
CT	236	32.91%
ET	239	33.33%
**ET plus mTORi after ET or CT (17 pts)**		
**ET plus mTORi after ET plus CDK4/6i (4 pts)**		
ET plus CDK 4/6i	61	8.51%
None	51	7.11%
Missing	130	18.13%
**Sites - second line**		
Bone only	117	16.32%
No visceral	103	14.37%
Visceral	314	43.79%
None therapy	51	7.11%
Missing	132	18.41%
**Number of lesions – second line**		
<3 sites	343	47.84%
≥3	199	27.75%
no therapy	51	7.11%
Missing	124	17.29%
**Number of lesions - second line**		
≤5 lesions	70	9.76%
<5 lesions	449	62.62%
No therapy	51	7.11%
Missing	147	20.50%
**Metastatic sites score- second line**		
<3 sites and ≤5 lesions	65	9.07%
≥3 sites and ≤5 lesions/ < 3 sites and > 5 lesions	260	36.26%
≥ 3 sites and >5 lesions	194	27.06%
No therapy	51	7.11%
Missing	147	20.50%
**Change sites between first and second line**		
No visceral → no visceral	106	14.78%
Visceral → visceral	366	51.05%
No visceral → visceral	62	8.65%
No therapy	51	7.11%
Missing	132	18.41%
**ECOG PS- second line**		
≤1	327	45.61%
>1	97	13.53%
No therapy	51	7.11%
Missing	242	33.75%
**Treatment strategies**		
ET plus CDK 4/6 → ET	19	2.65%
ET plus CDK 4/6 → CT	29	4.04%
Capecitabine 24 pts		
Nab-paclitaxel 2 pts		
Paclitaxel 1 pt		
Vinorelbine 2 pts		
ET followed by ET plus CDK 4/6	40	5.58%
ET followed by ET or CT	254	35.43%
CT followed by ET or CT	194	27.06%
No therapy	51	7.11%
Missing	130	18.13%
**Death events**		
Censored	269	37.52%
Uncensored	443	61.79%
Missing	5	0.70%
**PD 1 events**		
Censored	140	19.53%
Uncensored	575	80.20%
Missing	2	0.28%
**PD 2 events**		
Censored	63	8.79%
Uncensored	466	64.99%
Missing	188	26.22%
**PD 3 events**		
Censored	22	3.07%
Uncensored	135	18.83%
Missing	560	78.10%

Overall, 31% received first-line CT, 49% ET alone and 20% received ET plus CDK4/6i. In second-line, 33% of patients received CT, 33% ET alone and 8% ET plus CDK4/6i. In terms of treatment strategy, 27% of patients received CT followed by ET or CT, 35% received ET followed by CT or ET, 3% ET plus CDK4/6i followed by CT, 3% ET plus CDK4/6i followed by ET and 6% received ET followed by ET plus CDK4/6i (Table S1–S2).

With a median follow-up of 72.82 months (25th-75th percentile: 32.65–102.94 months), median PFS1 was 14.96 months (25th-75th percentile: 6.77–28.27), median PFS2 was 7.07 months (25th-75th percentile: 3.39–13.71) median PPS1 was 28.44 months (25th-75th percentile: 11.97–46.92), and median OS was 47.15 months (25th-75th percentile: 22.88–77.52).

### 3.2 Treatment strategies and tumor burden have a significant impact on survival outcomes

#### 3.2.1 PFS1

**Fig. 1:**
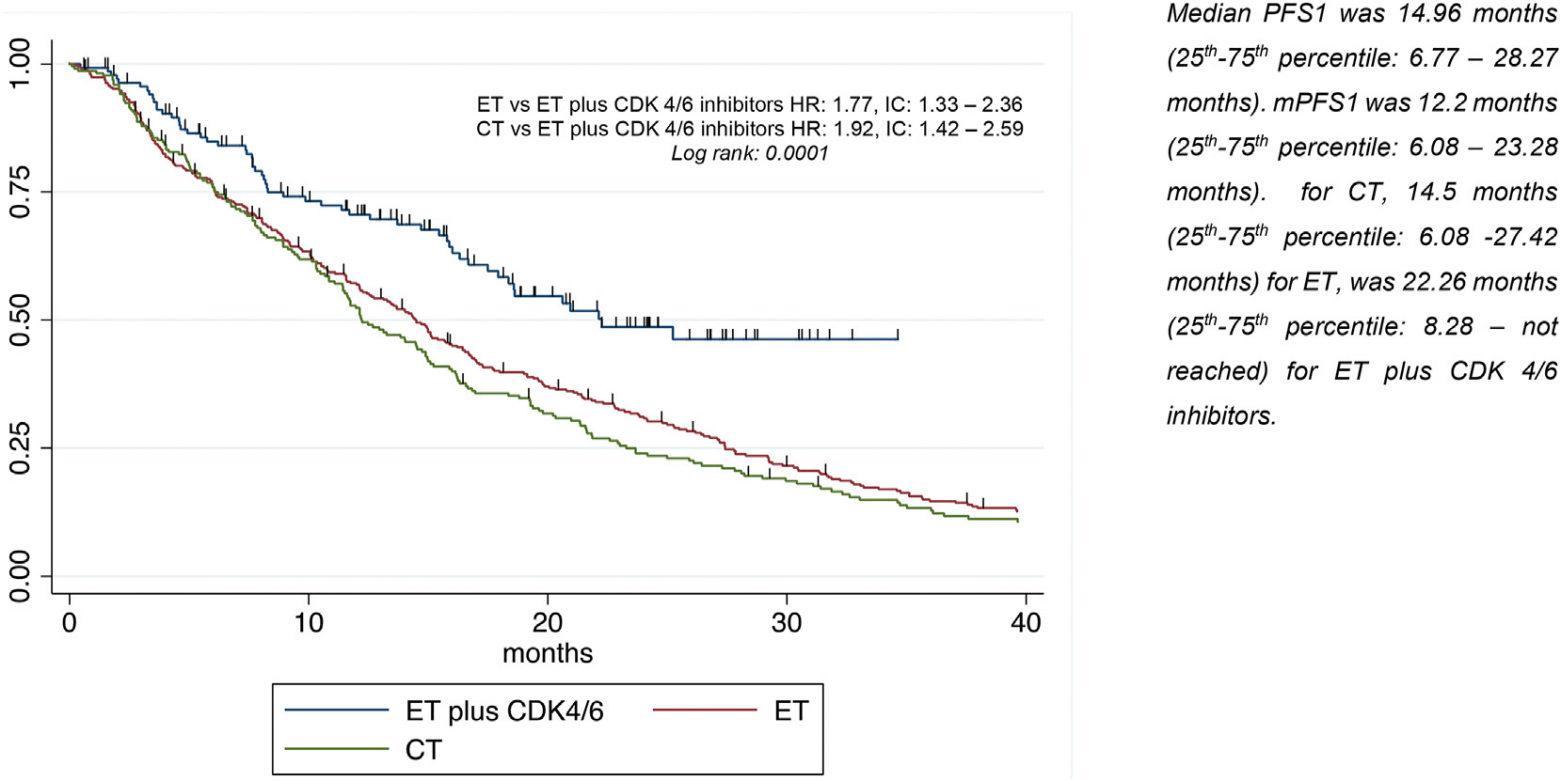
First line treatment-PFS1.

Univariate analysis for PFS1 showed that visceral disease, having ≥3 and >5 metastatic sites, ECOG PS > 1, Ki67 ≥ 14% and being previously exposed to ET were significantly associated with worse survival (Figure S1A-B-C). Conversely, *de novo* metastatic disease was associated with better PFS1. Patients treated with first-line ET (HR 1.77, p < 0.001, 95% C.I. 1.33–2.36) or CT (HR 1.92, p < 0.001, 95% C·I 1.42–2.59) experienced worse prognosis than patients treated with ET plus CDK4/6i. All variables retained their significance after multivariate analysis, in particular type of treatment received (HR 1.94, p < 0.001, 95%C.I. 1.37–2.73 for ET, HR 1.93, p < 0.001, 95%C.I. 1.36–2.75 for CT) (Table 2, Fig. 1).

**Table 2:** Univariate and multivariate analysis for PFS1

Variables	Univariate analysis			Multivariate analysis		
	HR	P	95 % CI	HR	P	95 % CI
**Age (years)**						
<45	1.00					
45-65	1.10	0.459	0.86 -1.40			
>65	1.26	0.063	0.99 – 1.62			
**BMI (kg/m**^**2**^**)**						
<25	1.00					
≥25	0.99	0.896	0.82 – 1.19			
**ET naive**						
Yes	**1.00**					
No	**1.21**	**0.028**	**1.02 – 1.43**	**1.45**	**0.001**	**1.17-1.79**
***De novo* metastatic disease**						
No	**1.00**					
Yes	**0.82**	**0.038**	**0.69 – 0.99**			
**Sites**						
Bone only	1.00					
Not visceral	1.17	0.188	0.93 – 1.48	1.26	0.132	0.93-1.71
Visceral	**1.29**	**0.009**	**1.07 – 1.56**	1.03	0.812	0.78-1.37
**Metastatic sites score**						
<3 sites and ≤5 lesions	**1.00**					
≥3 sites and ≤5 lesions/ < 3 sites and > 5 lesions	1.09	0.442	0.88 – 1.34	1.25	0.087	0.97-1.60
≥ 3 sites and >5 lesions	**1.48**	**0.001**	**1.16 – 1.87**	**1.64**	**0.003**	**1.18-2.28**
**ECOG PS-first line**						
≤1	**1.00**					
>1	**1.60**	**<0.001**	**1.27 – 2.01**	**1.36**	**0.025**	**1.04-1.77**
**ER**						
≤10 %	1.00					
>10 %	1.18	0.499	0.73 – 1.88			
**Ki-67 on primary tumor:**						
<14 %	**1.00**					
≥14 %	**1.37**	**0.002**	**1.13 -1.67**	**1.36**	**0.006**	**1.09-1.70**
**Endocrine responsiveness**						
No	1.00					
Yes	0.98	0.842	0.82 – 1.18			
**First-line therapy**						
ET plus CDK 4/6 inhibitors	**1.00**					
ET	**1.77**	**<0.001**	**1.33 – 2.36**	**1.94**	**<0.001**	**1.37-2.73**
CT	**1.92**	**<0.001**	**1.42 – 2.59**	**1.93**	**<0.001**	**1.36-2.75**

#### 3.2.2 PFS2

Visceral involvement (HR 1.36, p0.005, 95%C.I. 1.10–1.68), ECOG PS > 1 at second-line (HR 1.81, p < 0.001, 95%C.I. 1.42–2.31), having ≥3sites and >5 lesions (HR 1.69, p = 0.001, 95%C.I. 1.24–2.30), Ki-67 ≥14% (HR1.41, p = 0.002, 95%C.I. 1.13–1.76) and treatments other than ET plus CDK4/6i (ET: HR1.65, p = 0.004, 95%C.I. 1.17–2.32; CT: HR 1.75, p = 0.001, 95%C.I. 1.24–2.47) were linked with worse prognosis after univariate analysis in terms of PFS2. After multivariate analysis, second-line treatment retained its significance, showing that ET or CT compared with ET/CDK4/6i combination were burdened by worse prognosis (respectively HR 1.67, p = 0.009, 95%C.I. 1.13–2.44 and HR 1.79, p = 0.003, 95% C.I. 1.22–2.61) (Table S3, Figure S2).

Median PFS1 for patients receiving first-line CDK4/6i was 22.26 months (25th-75th percentile: 8.28 – not reached). Conversely, median PFS2 for patients receiving second-line CDK4/6i was 12.26 months (25th-75th percentile: 5.16–22.32 months) (Figure S3).

#### 3.2.3 PPS

Univariate analysis for PPS1, showed a trend for better PPS in women treated with first-line ET as compared to ET plus CDK4/6i (HR: 0.63, C·I.: 0.37–1.06, p = 0.08) (Figure S4).

#### 3.2.4 OS

**Fig. 2:**
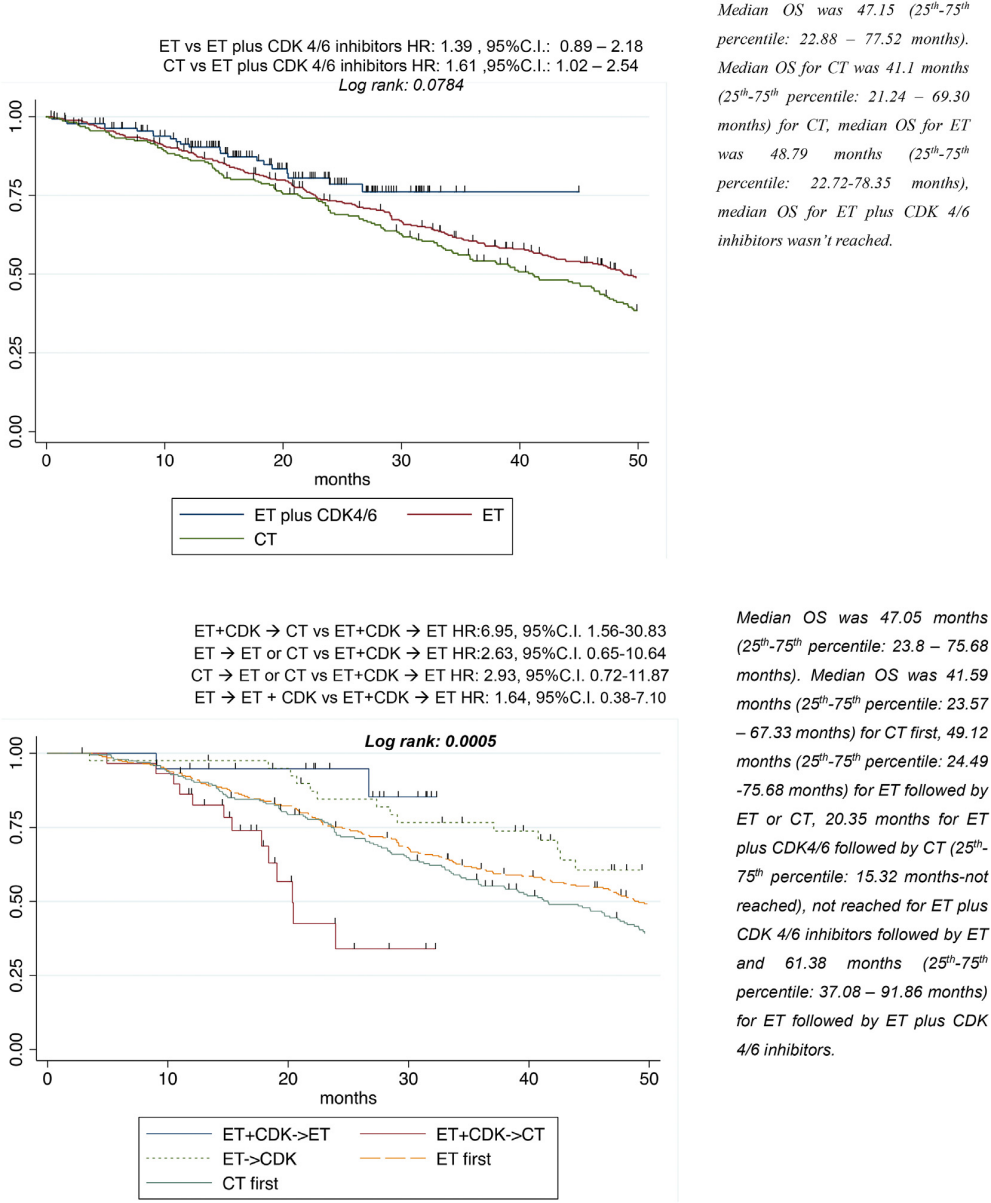
AFirst-line treatment-OS. B. Treatment strategies-OS

A trend towards better prognosis in terms of OS was observed in patients treated with first-line CDK4/6i (Table 3, Fig. 2A). In univariate analysis, ET followed by ET plus CDK4/6i, ET plus CDK4/6i followed by ET, ET followed by ET or CT and CT followed by CT or ET showed a better survival compared with ET plus CDK4/6i followed by CT. Results were confirmed in multivariate analysis (Table 3, Fig. 2B).

**Table 3:** Univariate and Multivariate analysis-OS

	Univariate analysis			Multivariate analysis (first-line therapy)			Multivariate analysis (strategies)		
Variables	HR	P	95 % CI	HR	P	95 % CI	HR	P	95 % CI
**Age (years)**									
< 45	1.00								
45-65	0.97	0.812	0.73 – 1.28						
> 65	1.26	0.111	0.95 – 1.66						
**BMI (kg/m**^**2**^**)**									
< 25	1.00								
≥ 25	0.90	0.292	0.73 – 1.10						
**ET naive**									
Yes	1.00								
No	1.18	0.097	0.97 – 1.43						
**CT naive**									
Yes	1.00								
No	1.03	0.746	0.85-1.25						
***De novo* metastatic disease**									
No	1.00								
Yes	0.93	0.494	0.75 –1.15						
**ER**									
≤10 %	1.00								
>10 %	0.70	0.171	0.43 – 1.16						
**Ki-67 on primary tumor:**									
<14 %	**1.00**								
≥14 %	**1.80**	**<0.001**	**1.42 – 2.28**	**1.91**	**<0.001**	**1.47-2.49**	**2.00**	**<0.001**	**1.49-2.68**
**Metastatic sites score**									
No	1.00								
Yes	1.10	0.369	0.89 – 1.37						
**Sites**									
Bone only	1.00								
No visceral	1.25	0.107	0.95 – 1.62	1.30	0.119	0.93-1.82	1.23	0.263	0.86-1.76
Visceral	**1.61**	**<0.001**	**1.30– 2.01**	1.20	0.262	0.87-1.66	0.99	0.960	0.70-1.41
**Sites score**									
<3 sites and ≤5 lesions	**1.00**								
≥3 sites and ≤5 lesions/ < 3 sites and > 5 lesions	**1.16**	0.224	0.91 – 1.48	1.20	0.206	0.90-1.59	1.28	0.112	0.94-1.74
≥ 3 sites and >5 lesions	**1.97**	**<0.001**	**1.51 – 2.56**	**1.60**	**0.009**	**1.13-2.28**	**1.83**	**0.002**	**1.24-2.68**
**ECOG PS**									
≤ 1	**1.00**								
> 1	**2.39**	**<0.001**	**1.89 – 3.02**	**2.07**	**<0.001**	**1.59-2.70**	**1.71**	**0.001**	**1.25-2.33**
**First-line therapy:**									
ET plus CDK 4/6 inhibitors	**1.00**								
ET	1.39	0.149	0.89 – 2.18	1.40	0.173	0.86-2.29			
CT	**1.61**	**0.042**	**1.02 – 2.54**	1.24	0.404	0.75-2.03			
**Treatment strategies**									
ET +CDK → ET	1.00								
ET+ CDK →CT	**6.95**	**0.011**	**1.56-30.83**				**5.05**	**0.035**	**1.12-22.72**
ET → ET or CT	**2.63**	**0.176**	**0.65-10.64**				2.35	0.235	0.57-9.61
ET→ ET + CDK	**1.64**	**0.508**	**0.38-7.10**				1.70	0.483	0.39-7.46
CT → CT or ET	**2.93**	**0.133**	**0.72-11.87**				2.05	0.318	0.50-8.39

### 3.2 Attrition rates across first-line treatment strategies

Attrition rate in the whole population was of 9.67% between first- and second-line treatment (PD1 observed in 575 patients, missing data in 130 patients and second-line treatment started in 536). Particularly, attrition rate was 7.31%, 8.25% and 17.24% for CT, ET alone and ET plus CDK4/6i, respectively. Moreover, probability to receive CT after ET plus CDK4/6i (treated in first- and second-line) was 52.80%. More in depth, the probability of receiving second- and third-line CT after ET plus CDK4/6i was 53.84% and 53.26%, respectively (Figure S5A, B).

Baseline characteristics at second-line that could influence the choice of CT or ET after CDK4/6i were analyzed. None of the variables considered, including rapidly progressive disease (defined as PFS ≤6 months at first-line) impacted on the choice of second-line treatment after CDK4/6i (Table S4).

## 4 Discussion

The addition of CDK4/6i in combination to ET has dramatically improved the clinical outcome of patients with luminal mBC, leading to rapid approval of these agents and recommendation of their use as the preferred first-line option by national and international guidelines [20,21].

The present study showed that first-line ET or CT alone were associated, in a real-world cohort, with worse PFS compared to ET plus CDK4/6i, with a 10 months difference in terms of mPFS1 for patients treated with combination therapy (mPFS1 12 months for CT, 14 months for ET and 22 months for ET plus CDK4/6i), consistently with what was observed in all main phase III studies [1,2,5,22].

Moreover, the present study showed a prolonged PFS2 in patients who received a CDK4/6i-based second-line as compared to ET or CT alone (12 months for ET plus CDK4/6i, 7 months for ET alone and 6 months for CT), consistently with PALOMA-3 trial which demonstrated an improvement in median PFS with the addition of Palbociclib to Fulvestrant of 9.5 months vs 4.6 months (HR 0.46, 95% CI 0.36–0.59, p < 0.0001)^23^.

Furthermore, MONALEESA-3 trials demonstrated a statistically significant benefit in OS with the combination of Ribociclib and ET. Consistently, in the phase III MONARCH-2 trial, Abemaciclib improved OS when combined with Fulvestrant in second-line (HR 0.75; 95% CI: 0.606–0.945; p = 0.0137) [6,[24], [25], [26], [27], [28], [29]]. Although the present study showed a trend towards better prognosis in terms of OS in patients treated with first-line CDK4/6i, the median follow-up was not adequate to generate conclusive results.

Notably, the study showed a greater improvement of CDK4/6i in first-line rather than in second-line second (median PFS1 and PFS2 respectively 22 vs 12 months) (Fig. 2). Moreover, the rate of patients that developed visceral metastases after CDK4/6i was similar in both second-line CT and ET cohorts. Conversely, the largest proportion of patients who switched from non-visceral to visceral involvement were those receiving ET followed by ET or CT (P < 0.001) (Table S1).

Luminal mBC is a clinically and molecularly heterogeneous disease, and the specific mechanisms though which it develops resistance to ET and CDK4/6i are still not fully elucidated, hindering the definition of biology-driven sequence strategies [12,14,16,30].

In the analyzed cohort, 60% of patients received CT and 40% received ET after a first-line CDK4/6i-based strategy. About CT, 24 pts received capecitabine, 2 nab-paclitaxel, 2 vinorelbine and 1 paclitaxel. Interestingly, after CDK4/6i failure, a worse prognosis was observed in patients who received CT over ET (HR 6.95, p = 0.01). Because of the potential bias that could have favored CT assignment in patients with an impending visceral crisis and therefore with an inherently worse prognosis, a logistic regression model was developed to explore potential factors that could have influenced clinical decision-making in second-line after CDK4/6i. None of the analyzed factors seemed to influence the choice of second-line CT or ET. Consistently, a recent study showed that ET after a combination of ET and Palbociclib is still effective, leading to a long median PFS2 after disease progression to first-line treatment [31].

Results from a logistic regression analysis conducted on 525 patients who had previously received CDK4/6i suggested that patients treated with CT after CDK4/6i were more likely to have a rapidly progressive disease compared to ET, everolimus or subsequent CDK4/6i (OR 0.46, 0.59, and 0.48)[32]. These observations might support the choice of ET after disease progression on a CDK4/6i in clinical practice.

The choice of second-line agents depends on previous lines of treatment and, to date, current options in progressive disease after CDK4/6i are represented by mTOR inhibitors (e.g everolimus plus exemestane), ET alone, CDK4/6i, PI3K inhibitors or CT.

The PI3K/Akt/mTOR pathway potentially plays a crucial role in secondary endocrine resistance in luminal mBC and there is a strong biological rationale supporting PI3K/AKT/mTOR axis as a therapeutic target [33]. The phase III SOLAR 1 trial showed the efficacy and safety of the PI3K inhibitor Alpelisib plus Fulvestrant in patients previously treated with ET (median PFS was 11 months in the Alpelisib arm vs 5.7 months in the placebo arm; HR:0.65). These results highlighted the potential need for an upfront evaluation of the PIK3CA mutational status and support the use of Alpelisib in mutated mBC with secondary resistance to ET [34]. However, this type of treatment is not yet available in all countries, except in clinical trials or specific programs.

Noteworthy, the efficacy of combinations of ET with mTOR, PI3K, and CDK4/6 inhibitors, the biological interplay between these pathways and the sensitization of cancer mutant cells led to the design of clinical studies investigating triplet combinations [[2], [3], [4], [5],^22^,^23^,[35], [36], [37], [38]].

Moreover, *ESR1* mutations represent an established mechanism of acquired resistance to AI and, therefore, a combination of the same CDK4/6i with a more effective ET would overcome this resistance [39]. A recent study conducted on 16 mBC patients treated with Palbociclib and letrozole demonstrated that ESR1 mutations were significantly associated with worse PFS (3.3 vs 9.0 months; P = 0.038)^39^.

Consistently, the preliminary results of the PADA-1 study, suggested that the combination of Palbociclib with AIs could potentially overcome the initial impact of *ESR1* mutations, leading to the clearance of mutated clones which eventually reoccurred at progression [40]. Of note, alternative resistance mechanisms could affect the *ESR1* gene, such as epigenetic alterations, which can lead to early resistance and consequently to a dramatically shorter PFS1 under CDK4/6i [16].

Interestingly, the present study investigated attrition rate, a promising metric in evaluating the opportunity to receive a second-line treatment. Nuzzolese and Montemurro provided data on attrition rates in CDK4/6i trials. In the first-line setting, attrition rate ranged from 12% to 27% for ET and from 15% to 33% for CDK4/6i (PALOMA-2, PALOMA-3, MONARCH-2, MONARCH-3, MONALEESA-2, MONALEESA-3, MONALEESA-7 trials) [41]. In the present study, attrition rate was 7% for CT, 8% for ET and 17% for ET plus CDK4/6i after first-line therapy. Moreover, the probability to receive CT after CDK4/6i was 53%, regardless of the line of treatment, and approximately 54% and 53% after first- and second-line therapy, respectively.

This study, supports the hypothesis that second-line ET, combined with other targeted molecules, might potentially represent a feasible option after CDK4/6i failure, allowing to further postpone CT to later lines.

Noteworthy*,* this analysis contributes to the generation of real world data, which relevance has often been highlighted as an essential integration of randomized trials. Indeed, the use of data from the real-world scenario is garnering increased attention to provide information about clinical-relevant questions that cannot be answered using data from clinical trials [42].

Notwithstanding the intriguing insights, the present study has some limitations, such as the retrospective design and the small sample size. In particular, due to the paucity of patients receiving a CDK 4/6 Inhibitor (141 patients in the first line setting and 61 patients in the 2nd line setting) results need to interpreted with caution. Moreover, clinical decision-making is strongly influenced by patients’ and diseases’ characteristics, including age, PS, prior toxicities, potential adherence to treatment, previous treatment route of administration, and time to progression, with all of these characteristics being potentially sources of selection bias.

## 5 Conclusions

To date, first-line CDK4/6i-based therapies represent the gold standard in luminal mBC. The present study confirmed the significant impact of this strategy on a homogeneous real-world cohort. Moreover, the study suggested a preeminent role for ET after first-line CDK4/6i, supporting the concept of an ET switch to overcome potential resistance mechanisms and restore clinical response. Due to the retrospective nature of these results, further prospective studies will be needed to confirm these observations and to build the optimal treatment algorithms for luminal mBC.

## 6 Clinical practice points

• First-line (1st L) CDK4/6i-based therapies represent the standard treatment in luminal mBC. However, prospective head-to-head comparisons are still lacking for many 1st line options, and it is still crucial to define the best treatment strategy for both 1st and 2nd L.
• The present study confirmed the significant impact of 1st L CDK4/6i-based therapies on a homogeneous real-world cohort. Moreover, the study suggested a preeminent role for ET after first-line CDK4/6i, supporting the concept of an ET switch to overcome potential resistance mechanisms and restore clinical response.
• The second-line ET, combined with other targeted molecules, might potentially represent a feasible option after CDK4/6i failure, allowing to further postpone CT to later lines.

## Credit author statement

Debora Basile: Conceptualization, Methodology, Software, Formal analysis, Data curation, writing-review&editing, Visualization, Supervision, Project administration; Lorenzo Gerratana: Conceptualization, Methodology, Validation, Software, Visualization, Supervision; Carla Corvaja: Data curation, Writing – original draft, Visualization; Giacomo Pelizzari: writing-review&editing, Visualization, Supervision; Giorgia Franceschin: Data curation, Writing – original draft; Elisa Bertoli: Data curation, Visualization; Lorenza Palmero: Data curation, Visualization; Diego Zara: Data curation, Visualization; Martina Alberti: Data curation, Visualization; Silvia Buriolla: Data curation, Visualization; Lucia Da Ros: writing-review&editing, Visualization, Resources; Marta Bonotto: writing-review&editing, Visualization, Conceptualization; Mauro Mansutti: Visualization, Resources; Simon Spazzapan: Visualization, Resources; Marika Cinausero: writing-review&editing, Visualization, Resources; Alessandro Marco Minisini: writing-review&editing, Visualization, Resources, Supervision; Gianpiero Fasola: writing-review&editing, Visualization, Resources, Supervision; Fabio Puglisi: Methodology, writing-review&editing, Visualization, Resources, Supervision, Project administration

## Funding

No specific funding was available for this work.

## Declaration of competing interest

The authors declare no competing financial interests.
