# Supplementary material for: First- and second-line treatment strategies for hormone-receptor (HR)-positive HER2-negative metastatic breast cancer: A real-world study
Source: Breast. 2021 Mar 12;57:104–12. doi: 10.1016/j.breast.2021.02.015 (PMC8053791; doi:10.1016/j.breast.2021.02.015)
Supplement: Multimedia component 1 [file mmc1.docx]

**Figure S1B. Sites of Metastasis in terms of Progression Free Survival**

***Median PFS was 16.67 (25^th^-75^th^ percentile: 8.81 – 30.44 months) for bone only, 14.96 (25^th^-75^th^ percentile: 5.98 – 29.82 months) for not visceral disease, 12.53 (25^th^-75^th^ percentile: 5.75 – 24.99 months).***

**Figure S1B. Sites of Metastasis in terms of Post-Progression Survival**

***Median PPS was 36.49 (25^th^-75^th^ percentile: 18.9 – 53.79 months) for bone only, 28.83 (25^th^-75^th^ percentile: 16.27 – 48.16 months) for not visceral disease, 21.14 (25^th^-75^th^ percentile: 8.48 – 40.8 months).***

**Figure S1C. Sites of Metastasis in terms of Overall Survival**

***Median OS was 56.55 (25^th^-75^th^ percentile: 34.32 – 90.58 months) for bone only, 48.1 (25^th^-75^th^ percentile: 22.88 – 78.18 months) for not visceral disease, 35.67 (25^th^-75^th^ percentile: 18.35 – 64.9 months)***

**Figure S2. First line treatment for PFS2**

*Median PFS2 was 7.07 (25^th^-75^th^ percentile: 3.39 – 13.71 months). mPFS2 was 6.58 months ((25^th^-75^th^ percentile: 3.16 -11.84 months) for CT, 7.04 (25^th^-75^th^ percentile: 3.22 -12.99 months) for ET and 12.26 months (25^th^-75^th^ percentile: 5.16 -22.32 months) for ET plus CDK 4/6 inhibitors.*

ET vs ET plus CDK 4/6 inhibitors HR: 1.65, 95%C.I.: 1.17 – 2.32

CT vs ET plus CDK 4/6 inhibitors HR: 1.75, 95% C.I.: 1.24 – 2.47

*Log rank: 0.0052*

**Figure S3. PFS1 and PFS2 of treatment with CDK4/6 inhibitors**

*Median PFS1 for patients receiving CDK4/6 inhibitors in first line was 22.26 months (25^th^-75^th^ percentile: 8.28 – not reached), conversely median PFS2 for patients receiving CDK 4/6 inhibitors in second line was 12.26 months (25^th^-75^th^ percentile: 5.16 -22.32 months).*

**Figure S4. Treatment in terms of PPS**

*Median PPS1 for CT was 27.25 months (25^th^-75^th^ percentile: 10.55 – 43.69 months), for ET was 29.98 months (25^th^-75^th^ percentile: 13.02 -51.62 months) and for ET plus CDK 4/6 inhibitors was 16.31 months (25^th^-75^th^ percentile: 12.00– 22.09 months).*

.

ET vs ET plus CDK 4/6 inhibitors, HR: 0.63, C.I.: 0.37- 1.06

CT vs ET plus CDK 4/6 inhibitors HR: 0.74, C.I.: 0.44 – 1.26

*Log rank: 0.0912*

**Figure S5A. Attrition rate and probability to receive II line Figure S5B. Probability to receive CT after CDK4/6i after CDK4/6i**

**
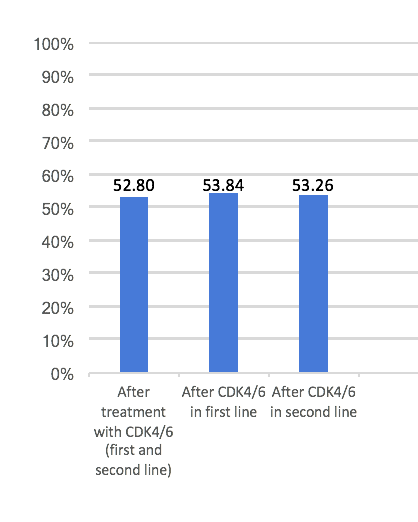

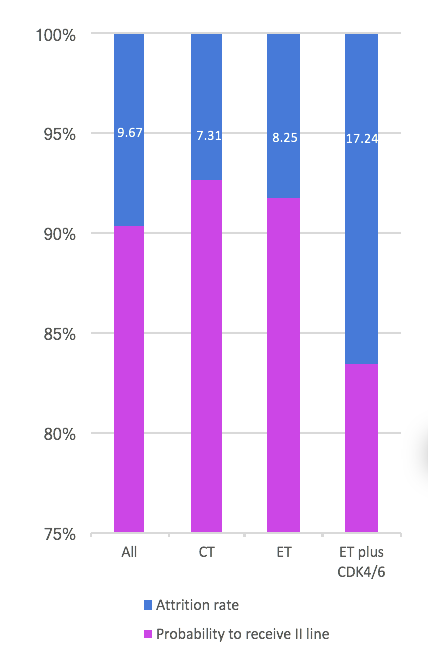
**
