# Supplementary material for: First- and second-line treatment strategies for hormone-receptor (HR)-positive HER2-negative metastatic breast cancer: A real-world study
Source: Breast. 2021 Mar 12;57:104–12. doi: 10.1016/j.breast.2021.02.015 (PMC8053791; doi:10.1016/j.breast.2021.02.015)
Supplement: Multimedia component 2 [file mmc2.docx]

**Table S1. Patient’s characteristics according to first-line treatment**

|  | **CT (N pts 224)** | **ET (N pts 352)** | **ET plus CDK4/6 (N pts 141)** | **P value** |
| --- | --- | --- | --- | --- |
| **Age**   - <45 - 45-65 - >65 - Missing | 46 (20.54 %)  119 (53.13 %)  57 (25.45 %)  2 (0.89 %) | 46 (13.07 %)  123 (34.94 %)  183 (51.99 %)  0 (0 %) | 14 (9.93 %)  72 (51.06 %)  55 (39.07 %)  (0 %) | **<0.001** |
| **Histotype**   - Ductal - Lobular - Other - Missing | 169 (75.44 %)  29 (12.95 %)  5 (2.23 %)  21 (9.36%) | 249 (70.74 %)  66 (18.75 %)  8 (2.27 %)  29 (8.24 %) | 95 (67.38 %)  32 (22.70%)  1 (0.71 %)  13 (9.22 %) | 0.120 |
| **Ki-67**   - <14 % - ≥14 % - Missing | 41 (18.30 %)  155 (69.20 %)  28 (12.50 %) | 104 (29.55 %)  183 (51.99 %)  65 (18.47 %) | 36 (25.53 %)  92 (65.25 %)  13 (9.22 %) | **0.001** |
| **CT naive**   - No - Yes | 114 (50.89 %)  110 (49.11 %) | 139 (39.49 %)  213 (60.51 %) | 59 (41.84 %)  82 (58.16 %) | **0.020** |
| **ET naive**   - No - Yes | 140 (62.50 %)  84 (37.50 %) | 216 (61.36 %)  136 (38.64 %) | 90 (63.83 %)  51 (36.17 %) | 0.365 |
| **ER**   - ≤ 10 % - > 10 % - Missing | 12 (5.36 %)  179 (79.91 %)  33 (14.73 %) | 9 (2.56 %)  295 (83.81 %)  48 (13.64 %) | 4 (2.84 %)  120 (85.11 %)  17 (12.06 %) | 0.165 |
| **BMI (kg/m^2^)**   - < 25 - ≥ 25 - Missing | 120 (53.57 %)  81 (36.16 %)  23 (10.27 %) | 101 (28.69 %)  167 (47.44 %)  84 (23.86 %) | 42 (29.79 %)  45 (31.91 %)  54 (38.30 %) | **<0.001** |
| ***De novo* metastatic disease**   - No - Yes - Missing | 155 (69.20 %)  68 (30.36 %)  1 (0.45 %) | 252 (71.59 %)  97 (27.56 %)  3 (0.85 %) | 96 (68.09 %)  43 (30.50 %)  2 (1.42 %) | 0.699 |
| **Endocrine responsiveness**   - No - Yes - Missing | 96 (42.86 %)  87 (38.84 %)  41 (18.30 %) | 141 (40.06 %)  120 (34.09 %)  91 (25.85 %) | 64 (45.39 %)  69 (48.94 %)  8 (5.67 %) | 0.538 |
| **Sites- first line**   - Bone only - No visceral - Visceral - Missing | 26 (11.61 %)  39 (17.41 %)  159 (70.98 %)  0 (0 %) | 153 (43.47 %)  83 (23.58 %)  115 (32.67 %)  1 (0.28 %) | 39 (27.66 %)  19 (13.48 %)  79 (56.03 %)  4 (2.84 %) | **<0.001** |
| **Metastatic sites number**   - <3 sites and ≤5 lesions - ≥3 sites and ≤5 lesions/ < 3 sites and > 5 lesions - ≥ 3 sites and >5 lesions - Missing | 34 (15.18%)  89 (39.73%)  80 (35.71%)  21 (9.38%) | 110 (31.25%)  164 (46.59%)  53 (15.06%)  25 (7.10%) | 33 (23.40%)  68 (48.23%)  33 (23.40%)  7 (4.96%) | **<0.001** |
| **ECOG PS-first line**   - ≤ 1 - > 1 - Missing | 140 (62.50 %)  29 (12.95 %)  55 (24.55 %) | 227 (64.49 %)  78 (22.16 %)  47 (13.35 %) | 127 (90.07 %)  1 (0.71%)  13 (9.22 %) | **<0.001** |
| **Menopause status**   - No - Yes - Missing | 71 (31.70 %)  135 (60.27 %)  18 (8.04 %) | 29 (8.24 %)  314 (89.20 %)  9 (2.56 %) | 32 (22.70%)  108 (76.60 %)  1 (0.71 %) | **<0.001** |
| **AST**   - ≤ 2.5 ULN - > 2.5 ULN - Missing | 161 (71.88%)  5 (2.23%)  58 (25.89%) | 202 (57.39%)  6 (1.70%)  144 (40.91%) | 107 (75.89%)  0 (0%)  0 (0%) | 0.199 |
| **GGT**   - ≤ 2.5 ULN - > 2.5 ULN - Missing | 90 (40.18%)  22 (9.82%)  112 (50.00 %) | 125 (35.51%)  12 (3.41%)  215 (61.08%) | 61 (43.26%)  6 (4.26%)  74 (52.48%) | **0.022** |
| **ALP**   - ≤ 2.5 ULN - > 2.5 ULN - Missing | 136 (60.71%)  14 (6.25%)  74 (33.04%) | 187 (53.13%)  10 (2.84%)  155 (44.03%) | 98 (69.50%)  2 (1.42%)  41 (29.08%) | **0.044** |
| **ALT**   - ≤ 2.5 ULN - > 2.5 ULN - Missing | 159 (70.98%)  7 (3.13%)  58 (25.89%) | 205 (58.24%)  3 (0.85%)  144 (40.91%) | 105 (74.47%)  3 (2.13%)  33 (23.40%) | 0.258 |
| **Site change between first and second line**   - No visceral 🡪 no visceral - Visceral-visceral - No visceral 🡪 visceral - Missing | 15 (6.70%)  164 (73.21%)  15 (6.70%)  30 (13.39%) | 84 (23.86%)  165 (46.88%)  44 (12.50%)  59 (16.76%) | 7 (4.96%)  37 (26.24%)  3 (2.13%)  94 (66.67 %) | **<0.001** |

**Table S2.** **Patient’s characteristics according to treatment strategies**

| **Variables** | **ET CDK 4/6**  **->ET (N pts 19)** | **ET CDK 4/6**  **->CT (N pts 29)** | **ET -> ET plus CDKi 4/6 (N pts 40)** | **ET ->CT or ET (N pts 254)** | **CT->ET or CT (N pts 196)** | **P value** |
| --- | --- | --- | --- | --- | --- | --- |
| **Age**   - < 45 - 45-65 - > 65 - Missing | 2 (10.53%)  9 (47.37%)  8 (42.11%)  0 (0%) | 3 (10.34%)  11 (37.93%)  15 (51.72%)  0 (0%) | 7 (17.50%)  9 (22.50%)  24 (60.00%)  0 (0%) | 32 (12.60 %)  98 (38.58%)  124 (48.82%)  0 (0%) | 44 (22.45%)  102 (52.04%)  48 (24.49%)  2 (1.02%) | **< 0.001** |
| **Histotype**   - Ductal - Lobular - Other - Missing | 12(63.16%)  4 (21.05%)  1 (5.26%)  2 (10.53%) | 14 (48.28%)  10 (34.48%)  0 (0%)  5 (17.24%) | 27 (67.50%)  9 (22.50%)  0 (0%)  4 (10.00%) | 182 (71.65%)  44 (17.32%)  7 (2.76%)  21 (8.27%) | 145 (73,98%)  28 (14.29%)  5 (2.55%)  18 (9.18%) | 0.147 |
| **Ki-67**   - <14 % - ≥14 % - Missing | 6 (31.58%)  13 (68.42%)  0 (0%) | 6 (20.69%)  20 (68.97%)  3 (10.34%) | 14 (35.00%)  21 (52.50 %)  5 (12.50 %) | 69 (27.17 %)  131 (51.57%)  54 (21.26 %) | 38 (19.39%)  132 (67.35%)  26 (13.27%) | 0.087 |
| **CT naive**   - No - Yes - Missing | 6 (31.58%)  13 (68.42%)  0 (0%) | 22 (75.86%)  7 (24.14%)  0 (0%) | 19 (47.50%)  21 (54.39 %)  0 (0%) | 108 (42.52%)  146 (57.48%)  0 (0%) | 98 (50.00%)  98 (50,00%)  0 (0%) | **0.006** |
| **ET naive**   - No - Yes - Missing | 9 (47.37%)  10 (52.63%)  0 (0%) | 27 (93.10 %)  2 (6.90 %)  0 (0 %) | 23 (57.50%)  17 (42.50 %)  0 (0 %) | 162 (63.78%)  92 (36.22%)  0 (0%) | 119 (60.71%)  77 (39.29%)  0 (0 %) | **0.006** |
| **ER**   - ≤ 10 % - > 10 % - Missing | 2 (10.53%)  16 (84.21%)  1 (5.26%) | 0 (0 %)  28 (96.55 %)  1 (3.45 %) | 2 (2.50 %)  36 (90.00 %)  3 (7.50 %) | 4 (1.57%)  210 (82.68%)  40 (15.75 %) | 9 (4.59%)  158 (80.61%)  29 (14.80%) | 0.102 |
| **BMI (kg/m^2^)**   - <25 - ≥25 - Missing | 5 (26.32%)  7 (36.84%)  7 (36.84%) | 9 (31.03%)  12 (41.38%)  8 (27.59%) | 6 (15.00%)  19 (47.50%)  15 (37.50%) | 75 (29.53%)  123 (48.43%)  56 (22.05%) | 103 (52.55%)  71 (36.22%)  22 (11.22%) | **<0.001** |
| **M de novo**   - No - Yes - Missing | 11 (57.89%)  8 (42.11%)  0 (0%) | 27 (93.10%)  2 (6.90%)  0 (0%) | 29 (72.50%)  11 (27.50%)  0 (0%) | 186 (73.23%)  66 (25.98 %)  2 (0.79%) | 133 (67.86%)  63 (32.14%)  0 (0%) | **0.035** |
| **Endocrine responsiveness**   - No - Yes - Missing | 10 (52.63%)  9 (47.37%)  0 (0%) | 12 (41.38%)  15 (51.72%)  2 (6.90%) | 22 (55.00%)  10 (25.00%)  8 (20.00%) | 92 (36.22 %)  95 (37.40 %)  67 (26.38 %) | 83 (42.35%)  77 (39.29%)  36 (18.37%) | 0.312 |
| **Sites**   - Bone only - Not visceral - Visceral - Missing | 6 (31.58%)  2 (10.53%)  11 (57.89%)  0 (0%) | 5 (17.24%)  5 (17.24%)  18 (62.07%)  1 (3.45%) | 17 (42.50%)  4 (10.00%)  18 (45.00%)  1 (2.50%) | 113 (44.49%)  67 (26.38%)  74 (29.13%)  0 (0%) | 21 (10.71%)  36 (18.37%)  139 (70.92%)  0 (0%) | **<0.001** |
| **Number of Sites**   - <3 sites and ≤5 lesions - ≥3 sites and ≤5 lesions/ < 3 sites and > 5 lesions - ≥ 3 sites and >5 lesions - Missing | 4 (21.05%)  8 (42.11%)  4 (21.05%)  3 (15.79%) | 6 (20.69%)  13 (44.83%)  9 (31.03%)  4 (3.45%) | 10 (25.00%)  19 (47.50%)  7 (17.50%)  4 (10.00%) | 82 (32.28 %)  119 (46.85 %)  34 (13.39 %)  19 (7.48 %) | 28 (14.29%)  80 (40.82%)  70 (35.71%)  18 (9.18%) | **<0.001** |
| **Menopause status**   - No - Yes - Missing | 5 (26.32%)  14 (73.68%)  0 (0%) | 2 (6.90%)  27 (93.10%)  0 (0 %) | 5 (12.50%)  33 (82.50 %)  2 (5.00%) | 19 (7.48 %)  228 (89.76%)  7 (2.76 %) | 68 (34.69%)  114 (58.16%)  14 (7.14%) | **<0.001** |
| **Site change**   - No visceral 🡪 no visceral - Visceral-visceral - No visceral 🡪 visceral - Missing | 4 (21.05%)  12 (63.16%)  1 (5.26%)  2 (10.53%) | 3 (10.34%)  24 (82.76%)  2 (6.90%)  0 (0%) | 11 (27.50%)  24 (60.00%)  3 (7.50%)  2 (5%) | 73 (28.74%)  141 (55.51%)  38 (14.96%)  2 (0.79%) | 15 (7.65%)  160 (81.63%)  15 (7.65%)  6 (3.06%) | **<0.001** |
| **ECOG PS - first line**   - ≤ 1 - > 1 - Missing | 18 (94.74%)  0 (0%)  1 (5.26%) | 26 (89.66%)  3 (10.34%)  0 (0 %) | 32 (80.00%)  2 (5.00%)  6 (15.00%) | 165 (64.96%)  48 (18.90%)  41 (16.14%) | 127 (64.80%)  21 (10.71%)  48 (24.49%) | **0.001** |

**Table S3. Univariate and Multivariate analysis-PFS2**

|  | **Univariate analysis** | | | **Multivariate analysis (therapy)** | | |
| --- | --- | --- | --- | --- | --- | --- |
| **Variables** | **HR** | **P** | **95 % CI** | **HR** | **P** | **95 % CI** |
| **Age**   - <45 - 45-65 - >65 | 1.00  0.88  0.88 | 0.341  0.343 | 0.67 – 1.15  0.67 – 1.15 |  |  |  |
| **ET naive**   - Yes - No | 1.00  1.16 | 0.127 | 0.96 – 1.40 |  |  |  |
| **Sites - second line**   - Bone only - Not visceral - Visceral | 1.00  1.21  **1.36** | 0.153  **0.005** | 0.93 – 1.56  **1.10– 1.68** | 1.24  1.06 | 0.164  1.652 | 0.91-1.69  0.80-1.42 |
| **ECOG PS - second line**   - ≤1 - >1 | **1.00**  **1.81** | **<0.001** | **1.42 – 2.31** | **1.68** | **<0.001** | **1.27-2.23** |
| **ER**   - ≤10 % - >10 % | 1.00  0.73 | 0.280 | 0.41 – 1.30 |  |  |  |
| **Hormone responsiveness**   - No - Yes | 1.00  1.22 | 0.063 | 0.99- 1.50 |  |  |  |
| **Metastatic sites number second line**   - <3 sites and ≤5 lesions - ≥3 sites and ≤5 lesions/ < 3 sites and > 5 lesions - ≥ 3 sites and >5 lesions | **1.00**  1.14  **1.69** | 0.405  **0.001** | 0.84 – 1.53  **1.24 – 2.30** | **1.47**  **2.06** | **0.038**  **<0.001** | **1.02-2.11**  **1.40-3.04** |
| **Ki 67:**   - <14 % - ≥14 % | **1.00**  **1.41** | **0.002** | **1.13-1.76** |  |  |  |
| **Therapy**   - ET plus CDK 4/6 inhibitors - ET - CT | **1.00**  **1.65**  **1.75** | **0.004**  **0.001** | **1.17 – 2.32**  **1.24 – 2.47** | **1.67**  **1.79** | **0.009**  **0.003** | **1.13-2.44**  **1.22-2.61** |

**Table S4. Logistic for second-line: determinats for the choice of second line (ET or CT) after CDK4/6**

|  | **Univariate analysis** | | |
| --- | --- | --- | --- |
| **Variables** | **OR** | **P** | **95 % CI** |
| **Age**   - < 45 - 45-65 - > 45 | 1.00  0.81  1.25 | 0.840  0.826 | 0.11-5.99  0.17-9.09 |
| **Histotype**   - Ductal - Lobular | 1.00  2.14 | 0.283 | 0.53 -8.62 |
| **Ki 67:**   - <14 % - ≥14 % | 1.00  1.54 | 0.525 | 0.41 -5.82 |
| **Endocrine responsiveness**   - No - Yes | 1.00  1.39 | 0.585 | 0.43-4.51 |
| **Site change**   - No visceral - Visceral- visceral - No visceral-visceral | 1.00  2.67  2.67 | 0.244  0.497 | 0.51-13.88  0.16-45.14 |
| **Number of lesions, second-line**   - ≤ 5 - > 5 | 1.00  0.75 | 0.820 | 0.06-8.90 |
| **Sites - second-line**   - Bone only - No visceral - Visceral | 1.00  3.33  2.05 | 0.288  0.394 | 0.36 – 30.70  0.39 – 10.70 |
| **ECOG PS – second line**   - ≤1 - >1 | 1.00  0.63 | 0.749 | 0.04 –10.75 |
| **PFS1 ≤6 months**   - **No** - **Yes** | 1.00  0.71 | 0.671 | 0.15-3.37 |
